# Investigating Factors Influencing Medical Practitioners’ Resistance to and Adoption of Internet Hospitals in China: Mixed Methods Study

**DOI:** 10.2196/46621

**Published:** 2023-07-31

**Authors:** Wenhao Deng, Tianan Yang, Jianwei Deng, Ran Liu, Xueqin Sun, Gang Li, Xinmei Wen

**Affiliations:** 1 School of Management and Economics, Beijing Institute of Technology Beijing China; 2 Sustainable Development Research Institute for Economy and Society of Beijing Beijing China; 3 School of Public Health and Management, Wenzhou Medical University Wenzhou China; 4 Department of Medical Insurance Management, Peking Union Medical College Hospital, Chinese Academy of Medical Science and Peking Union Medical College Beijing China; 5 TongJi Hospital, TongJi Medical College, Huazhong University of Science and Technology Wuhan China; 6 Department of Neurology, Xuanwu Hospital, Capital Medical University Beijing China

**Keywords:** internet hospital, conservation of resource theory, medical practitioner, Unified Theory of Acceptance and Use of Technology, technostress, resistance to change, behavioral intention to use

## Abstract

**Background:**

The swift shift toward internet hospitals has relied on the willingness of medical practitioners to embrace new systems and workflows. Low engagement or acceptance by medical practitioners leads to difficulties in patient access. However, few investigations have focused on barriers and facilitators of adoption of internet hospitals from the perspective of medical practitioners.

**Objective:**

This study aims to identify both enabling and inhibiting predictors associated with resistance and behavioral intentions of medical practitioners to use internet hospitals by combining the conservation of resources theory with the Unified Theory of Acceptance and Use of Technology and technostress framework.

**Methods:**

A mixed methods research design was conducted to qualitatively identify the factors that enable and inhibit resistance and behavioral intention to use internet hospitals, followed by a quantitative survey-based study that empirically tested the effects of the identified factors. The qualitative phase involved conducting in-depth interviews with 16 experts in China from June to August 2022. Thematic analysis was performed using the qualitative data analysis software NVivo version 10 (QSR International). On the basis of the findings and conceptual framework gained from the qualitative interviews, a cross-sectional, anonymous, web-based survey of 593 medical practitioners in 28 provincial administrative regions of China was conducted. The data collected were analyzed using the partial least squares method, with the assistance of SPSS 27.0 (IBM Corp) and Mplus 7.0 (Muthen and Muthen), to measure and validate the proposed model.

**Results:**

On the basis of qualitative results, this study identified 4 facilitators and inhibitors, namely performance expectancy, social influence, work overload, and role ambiguity. Of the 593 medical practitioners surveyed in the quantitative research, most were female (n=364, 61.4%), had a middle title (n=211, 35.6%) or primary title (n=212, 35.8%), and had an average use experience of 6 months every year. By conducting structural equation modeling, we found that performance expectancy (β=−.55; *P*<.001) and work overload (β=.16; *P*=.005) had the most significant impact on resistance to change. Resistance to change fully mediated the influence of performance expectancy and partially mediated the influences of social influence (variance accounted for [VAF]=43.3%; *P*=.002), work overload (VAF=37.2%; *P*=.03), and role ambiguity (VAF=12.2%; *P*<.001) on behavioral intentions to use internet hospitals. In addition, this study found that the sex, age, professional title, and use experience of medical practitioners significantly moderated the aforementioned influencing mechanisms.

**Conclusions:**

This study investigated the factors that facilitate or hinder medical practitioners’ resistance to change and their behavioral intentions to use internet hospitals. The findings suggest that policy makers avoid the resistance and further promote the adoption of internet hospitals by ensuring performance expectancy and social influence and eliminating work overload and role ambiguity.

## Introduction

### Background

The transition to internet hospitals in the digital era was designed to provide convenient and contactless health care to patients [1,2]. Internet hospitals encompass a wide range of portals, applications, or platforms that extend beyond traditional web-based health, mobile health, or telemedicine services. Unlike these services, which are primarily defined as health care provisions, internet hospitals offer a comprehensive array of functions, including appointment scheduling, consultation, treatment, and dispensing services [3,4]. After the State Council recognized internet hospitals for the first time in China in 2018, this new digital health care service model has, to some extent, relieved pressure on offline health care [5] and corrected inappropriate medical behaviors [6,7], especially during the COVID-19 epidemic. However, lower fees [8], increased workload [9], and other potential risks [10,11] for medical practitioners caused by internet hospitals were gradually exposed. In 2021, less than 10% of internet hospitals were able to achieve effective and sustainable operations. Therefore, with the restoration of offline health services, some medical practitioners may resist delivering digital health services via internet hospitals.

Resistance to change by medical practitioners is one of the main problems hindering the success of transition to health information systems [12,13] or applications [14,15] for a long time. Simultaneously, the behavioral intentions of medical practitioners to use health information systems or applications refer to their subjective inclination or the probability of adopting them, which plays a crucial role in determining their actual use behavior [16,17]. Recent research also calls for avoiding resistance and boosting the adoption of medical practitioners for internet hospitals [18,19]. However, few investigations have focused on barriers and facilitators of internet hospital adoption from the perspective of medical practitioners rather than patients [20,21]. In addition, prior work in internet hospitals pays less attention to resistance of medical practitioners, which would lead to many undesirable outcomes, such as lower use intentions [22], lower performance [23,24], and poorer behavioral decisions [12]. Given that the concept of internet hospitals is an emerging field that has not been fully researched, relevant research is rather scarce and fragmented. It is therefore difficult to use general findings from existing studies to extrapolate the antecedents of resistance or adoption by medical practitioners or to formulate hypotheses directly using the existing body of theory.

### Objectives

To provide both theoretical and empirical evidence, we conducted a mixed methods design [25] through an exploratory qualitative study and a validated quantitative survey across national and regional internet hospitals in China. Drawing on the conservation of resources (COR) theory, this study proposes a research model to answer the following research questions from the perspective of what, how, why, and who:

1. What are the predicting factors that determine medical practitioners’ resistance to change induced by using internet hospitals?
2. What are the predicting factors that determine medical practitioners’ behavioral intention to use internet hospitals?
3. Why and how do these predicting factors affect behavioral intention to use internet hospitals through resistance to change?
4. How do the boundary conditions of resource gain and depletion affect the resistance and intention to use internet hospitals?

### Theoretical Background

We built our conceptual framework on insights from the COR theory, which was developed by Hobfoll [26] and was based on the idea that anyone has a desire to acquire, maintain, and preserve their own resources [27]. Existing research emphasizes the role of the COR theory in the implementation and use of health information systems to help policy makers understand how individuals make decisions based on an assessment of resource depletion or resource gain [28]. Therefore, this study uses 2 corollaries of the COR theory as the theoretical basis for examining the predictors, mechanisms, and boundary conditions of medical practitioners’ resistance to change and intention to use, including the “resource depletion approach” and the “resource gain approach.”

On the one hand, the COR theory suggests that when individuals feel an increase in resources, they will release negative emotions and the rate of resource gain will increase, creating a virtuous resource gain approach. In the field of information systems, the Unified Theory of Acceptance and Use of Technology (UTAUT) provides 4 positive resources that determine behavioral intentions of users: performance expectations, effort expectations, social influence, and facilitating conditions [29]. These resources have been used globally in a variety of technological applications such as telemedicine in Nigeria [30]; electronic health records in Canada, Malaysia, and Bangladesh [13,31,32]; and the BeIRAI health care project in Flanders [33]. The definition of each construct is presented in Multimedia Appendix 1 [29,34]. Accordingly, the use of the UTAUT to explain the acceptance of emerging health care applications by medical practitioners has become an important topic in recent research, including artificial intelligence–based medical diagnosis support systems [35], electronic health records [36], mobile nursing apps [37], and mobile health care applications [15].

On the other hand, the COR theory suggests that when individuals perceive that resources are likely to be lost or have been lost, psychological stress and strain are created and resources are more likely to be lost, creating a vicious resource depletion approach. In the field of information technology, the adverse psychological, physiological, and behavioral effects of modern information technology on users’ work are known as technostress [38], which includes 4 main dimensions: work overload, role ambiguity, invasion of privacy, and work-family conflict [34]. The definition of each construct is presented in Multimedia Appendix 1. As a stress response, the technostress framework could substantially trigger employees’ intention to leave, job burnout, and feelings of job maladjustment [39,40]. Although research on the impact of technostress on medical practitioners is still in the preliminary stages of exploration, there is evidence that technostress on telemedicine and electronic health records can negatively impact the performance of medical practitioners in using technology or systems [41,42].

Finally, although researchers have encouraged the use of demographic factors as moderators to fully understand how medical practitioners’ adoption of or resistance to health information technologies and systems adapts to various contexts [29,43], not many relevant studies have been conducted. A few studies have found that older and more experienced physicians are more resistant to the adoption of electronic medical records systems [13,44]. In addition, the experience of using telemedicine services has a prominent impact on service use and satisfaction of physicians [45]. Considering this research gap, this study explores the moderating role of sex, age, professional title, and use experience in the relationship between all facilitators and inhibitors of resistance to change, based on qualitative interviews and quantitative investigations of internet hospitals.

In summary, the UTAUT and technostress framework, guided by the COR theory, served as the theoretical basis for explaining resource gain and resource depletion among medical practitioners, respectively, and helped us explore the mechanism behind when, how, and why they adopt or resist new health care information systems or applications. We argue that the internet hospital, as an extension of the telemedicine and traditional hospital models [4], also applies to the UTAUT model and technostress framework. Therefore, this study uses a mixed methods approach [25] to qualitatively identify the factors that enable and inhibit resistance and behavioral intention to use internet hospitals, followed by a quantitative survey-based study that empirically tests the effects of the identified factors.

## Methods

### Qualitative Approach

#### Overview

This study used a combination of purposive sampling and snowball sampling to select interviewees to ensure that the results were representative of the views and insights regarding the use of internet hospitals. A semistructured interview guide was developed by applying the COR theory to construct the influencing approaches into a resource gain approach and a resource depletion approach [27].

#### Participant Recruitment

In-depth interviews were conducted with 16 experts in this study from June to August 2022, including leaders of government agencies involved in the development of internet hospital policies, managers of health care institutions where internet hospitals were well established, high-level physicians directly using internet hospitals, key scholars involved in related research, and third-party companies or technical leaders assisting with the construction of internet hospitals. Monetary incentives were provided to encourage qualified interviewees. The background information of the respondents is presented in Table 1.

**Table 1:** Overview of interview data sources (N=16).

Code	Sex	Age (years)	Designation	Work experience (years)	City
R1	Female	52	Hospital office director	15	Beijing
R2	Male	36	Deputy director of medical insurance administration	11	Beijing
R3	Male	46	Head of outpatient department	23	Beijing
R4	Male	38	Director of hospital information center	15	Beijing
R5	Male	42	General manager of Beijing Tianjin Hebei region	19	Beijing
R6	Male	46	Associate chief physician	22	Beijing
R7	Male	35	Research associate in telemedicine hub	10	Beijing
R8	Female	53	Director of hospital information center	30	Beijing
R9	Female	39	Chief physician	15	Nanchang
R10	Female	35	Chief physician	7	Nanchang
R11	Female	35	Chief physician	5	Beijing
R12	Male	39	Hospital office director	17	Wuhan
R13	Female	46	Professor and doctoral supervisor	18	Wuhan
R14	Male	36	Medical department staff	11	Xiamen
R15	Male	46	Director of hospital information center	26	Wenzhou
R16	Male	39	Associate chief physician	9	Hangzhou

#### Procedure

Each interview process consisted of a facilitator, 2 instructors, and 2 professional notetakers, and the interview lasted between 30 and 45 minutes. The interview outline was dynamically adapted and refined according to the characteristics of the interviewees and the results of previous interviews. Audio recordings were transcribed using technical means after the interviews. We used thematic analysis to analyze the data collected from the interviews and obtained thematic codes using the qualitative data analysis software NVivo version 10 (QSR International). Finally, the open codes were summarized and reviewed by a panel of 5 experts with multidisciplinary backgrounds in medicine, natural sciences, and public policy. Instances where agreement could not be reached in coding were excluded, and a consensus was finally reached based on an intercoder reliability score of at least 92% [46]. Following coding, analysis, and distillation of qualitative interviews, we ultimately identified the facilitators and inhibitors influencing medical practitioners’ behavioral intentions and resistance toward using internet hospitals.

### Quantitative Approach

#### Study Design and Measures

We integrated established scales with the COR theory to create a web-based questionnaire (Multimedia Appendix 2). All questions were briefly modified in relation to the theme of internet hospitals, and all latent variables were scored on a Likert scale of 1 to 5. In addition, the study examined sex, age, professional title, and use experience as moderating variables in a separate question.

#### Sample and Data Collection

This study conducted a cross-sectional, anonymous, web-based survey of medical practitioners (aged >18 years) in 28 provincial administrative regions of China through a professional data collection platform (Credamo) in China. The platform's sample database included 2.8 million Chinese members with confirmed personal information and a range of socioeconomic backgrounds. This study distributed questionnaires to internet hospital platforms nationwide, including internet hospitals founded by public hospitals, private hospitals, digital health care enterprises, and local governments.

This study used 3 research design processes to ensure the quality of responses to the web-based survey. First, 2 validation questions were used to ensure that participants were aware of the definition and format of an internet hospital and that they had participated in using it as a physician before the formal survey began. The participants who did not pass the validation questions were asked to end their responses. Second, to ensure the reliability of our data, we implemented a screening process to exclude any abnormal response times. Questionnaires with unusually short or long completion times were removed. Specifically, we excluded questionnaires that were completed in less than 3 minutes or more than 20 minutes. The rationale behind this decision was to eliminate potential cases of rushed or careless responses, as well as instances of prolonged distractions or fatigue during the survey. Finally, each mobile device was only allowed to access the web-based questionnaire once, and participants were asked to provide the last 4 digits of their mobile phone number to avoid duplicate responses.

A total of 800 questionnaires were distributed for this web-based survey, and 593 (74.1% valid response rate) valid questionnaires were returned. A payment of Chinese RMB 5 Yuan (US $0.75) was sent to the participants who met the requirements. All data were stored in Credamo’s web-based servers and were password protected.

#### Data Analysis

The partial least squares method, a statistical analysis technique based on structural equation modeling, was used to measure and validate the proposed model. The collected data were inputted into SPSS 27.0 (IBM Corp) and Mplus 7.0 (Muthen and Muthen) software to conduct the required statistical analyses. The measurement model was evaluated by examining the internal reliability, convergent validity, discriminant validity, and collinearity statistics. The model fit tests were conducted before the mediation models and moderated mediation models, including the ratio between chi-square and the *df*, comparative fit index, Tucker-Lewis index, root mean square error of approximation, and Akaike information criterion. In this study, 5000 bootstrap samples were used to test the moderated mediation model to determine whether the level of a moderated variable was important. Mediation was proven if the CI of the indirect effect did not include 0. When substantial mediation was established, conditional indirect effect procedures were used to determine whether mediation depended on the level of the proposed moderator (ie, age, sex, professional title, and use experience).

### Ethics Approval

All procedures performed in this study involving human participants were approved by the First Affiliated Hospital of Xiamen University, China (KYX2016007). In addition, the participants were informed that their participation was voluntary, that they were free to decline or discontinue their participation at any time, and that their responses were processed anonymously and only used for research purposes. No person-identifying information was collected.

## Results

### Qualitative Findings

In terms of the resource gain approach, performance expectations and social influence in the UTAUT model were hypothesized to alleviate resistance to change and promote behavioral intentions. For example, a young physician said, “my superiors encouraged me to improve my reputation through internet hospitals,” whereas an older physician said, “I am more concerned about whether internet hospitals give me enough compensation as offline diagnosis and treatment.” In particular, this study removed effort expectancy and facilitating condition constructs from the UTAUT. As a new health care platform, internet hospitals were not as difficult to operate as medical technology; therefore, there was no question of a difference in the cost of effort and facilitating condition to use it. For example, the head of the hospital information department said, “Physicians were able to learn the operation of the internet hospitals very quickly and with high quality to the extent that they did not need additional training from the hospital.”

On the contrary, in terms of resource depletion approach, we suggest that work overload and role ambiguity in the technostress framework will predict the psychological and behavioral responses of internet hospitals by medical practitioners. For example, the specialist physicians interviewed said, “Internet hospitals have taken away from my fragmented time.” Similarly, medical practitioners who had just started using the internet hospitals said, “Internet hospital has disrupted my work rhythm because I don’t know whether I should prioritize online or offline demand.” In particular, according to the interviews, this study removed the invasion of privacy and work-family conflict constructs in the technostress framework. Because most internet hospitals relied on the hospital’s internal systems, using internet hospitals did not conflict substantially with family matters or infringe personal privacy of physicians. For example, the hospital administrator said, “We did not require physicians to use the internet hospitals for office work at home because the operating platform of the internet hospitals could only be connected from the office.”

**Figure 1:**
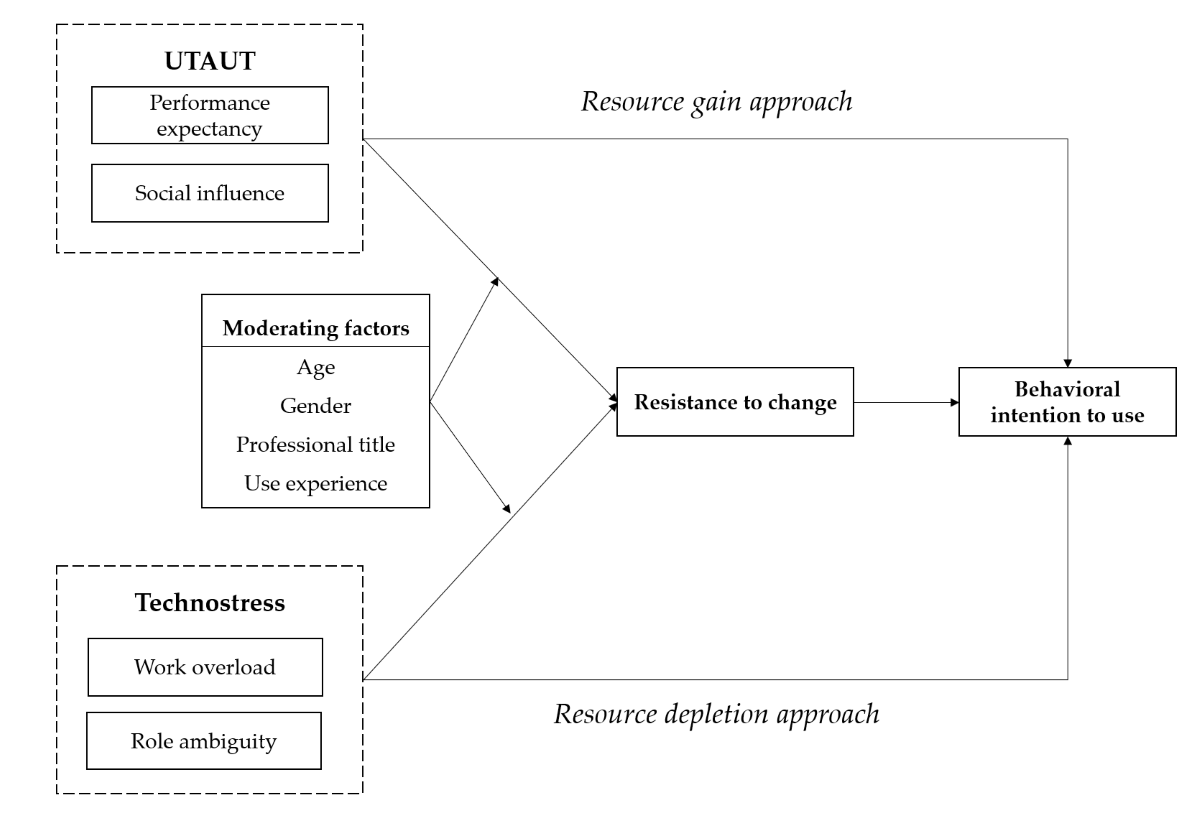
Conceptual model of resistance and adoption of internet hospitals. UTAUT: Unified Theory of Acceptance and Use of Technology.

On the basis of the results, this study identified 4 factors that either enable or hinder the adoption of internet hospitals: performance expectancy, social influence, work overload, and role ambiguity. These perceptions of gaining or losing resources can impact medical practitioners’ resistance to change and their behavioral intentions to use internet hospitals. In conjunction with the derivations from the theoretical background, we constructed a conceptual model that reflects these factors, as shown in Figure 1.

### Quantitative Findings

#### Profile and Characteristics of Participants

The demographic characteristics of respondents presented in Table 2 reveal that most were female (364/593, 61.4%) and had a middle title (211/593, 35.6%) or primary title (212/593, 35.8%). In terms of age and usage experience, the participants were aged 19 to 63 (mean 33.9, SD 8.8) years and used internet hospitals for an average of 6 (SD 3.4; range 1-12) months every year, respectively.

**Table 2:** Sample description (N=593).

Demographic characteristics			Participants, n (%)
**Sex**			
	Male	229 (38.6)	
	Female	364 (61.4)	
**Professional title^a^**			
	Senior	18 (3)	
	Deputy senior	108 (18.2)	
	Middle	211 (35.6)	
	Primary	212 (35.8)	
	None	44 (7.4)	

^a^The professional titles of medical practitioners include the primary title (eg, medical nurse, physician, or resident), middle title (eg, attending physician), deputy senior title (eg, associate chief physician), senior title (eg, chief physician), and no title.

#### Measurement Model Analysis

The measurement model was evaluated by examining its internal reliability, convergent validity, and discriminant validity. The estimated Cronbach α values ranged from .87 to .92, which were above the cutoff point of .85; the composite reliability values ranged from 0.91 to 0.94, which were above the cutoff point of 0.70 (Table 3). In addition, the calculated construct loadings ranged from 0.76 to 0.94 (Multimedia Appendix 2) and average variance extracted ranged from 0.72 to 0.85 (Table 3), which were above the cutoff point of 0.50 [47]. Therefore, the conditions for internal reliability and convergent validity were satisfied in this study.

Moreover, discriminant validity was measured by the square root of the average variance extracted and cross-loading matrix. The square root of the average variance extracted for each construct was larger than its correlation with the other constructs (Table 4), implying adequate discriminant validity.

Finally, to test multicollinearity issues, we estimated the variance inflation factor values of all the constructs, which were found to be <5 (Table 5). Therefore, the constructs in this model were not influenced from multicollinearity issues [48].

**Table 3:** Reliability and validity statistics.

Latent variables	Cronbach α	Composite reliability	Average variance extracted
Behavioral intention to use	.89	0.93	0.81
Resistance to change	.87	0.91	0.72
Performance expectancy	.88	0.92	0.75
Social influence	.88	0.93	0.81
Work overload	.91	0.94	0.85
Role ambiguity	.92	0.94	0.81

**Table 4:** Correlation analysis and discriminant validity.

Constructs	Behavioral intentions to use internet hospitals	Resistance to change	Performance expectancy	Social influence	Work overload	Role ambiguity
Behavioral intentions to use internet hospitals	0.90^a^	—^b^	—	—	—	—
Resistance to change	−0.77	0.85^a^	—	—	—	—
Performance expectancy	0.66	−0.71	0.87^a^	—	—	—
Social influence	0.69	−0.71	0.58	0.89^a^	—	—
Work overload	−0.71	0.70	−0.59	−0.67	0.92^a^	—
Role ambiguity	−0.74	0.63	−0.58	−0.54	0.64	0.89^a^

^a^The diagonal values are the square root of the average variance extracted.

^b^Not applicable.

**Table 5:** Collinearity statistics.

Constructs	Values, mean (SD)	Behavioral intentions to use internet hospitals^a^	Resistance to change^a^
Resistance to change	2.296 (0.716)	3.150	N/A^b^
Performance expectancy	3.717 (0.444)	2.181	1.850
Social influence	4.188 (0.753)	2.302	2.024
Work overload	2.277 (0.891)	2.509	2.346
Role ambiguity	2.217 (0.731)	1.974	1.920

^a^The values shown in the columns are variance inflation factors.

^b^N/A: not applicable.

#### Mediation Model Analysis

The partial least squares bootstrapping procedure with 5000 subsamples was performed to determine the statistical significance and relevance of the path coefficients in the structural model. The mediation model fit index was appropriate (Multimedia Appendix 3). We then summarized the results of bootstrapping, that is, path coefficient (β) and significance value (*P* value) of the paths in the proposed model (Table 6). Among the positive and negative predictors, performance expectancy (β=−.55; *P*<.001) and work overload (β=.16; *P*=.005) had the most significant impact on resistance to change, respectively. As expected, resistance to change had a significant negative impact on behavioral intention to use internet hospitals.

**Table 6:** Path analysis.

Path	β	*P* value
Performance expectancy→behavioral intentions to use	.101	.19
Performance expectancy→resistance to change	−.551	<.001
Social influence→behavioral intentions to use	.154	.002
Social influence→resistance to change	−.354	<.001
Work overload→behavioral intentions to use	−.089	.03
Work overload→resistance to change	.158	.005
Role ambiguity→behavioral intentions to use	−.343	<.001
Role ambiguity→resistance to change	.144	.03
Resistance to change→behavioral intentions to use	−.332	<.001

Mediation analysis was performed to assess the mediating effect of resistance to change in the proposed model. As shown in Table 7, all indirect effects were significant, as all 95% CIs did not contain 0. The direct effect between performance expectancy and behavioral intentions was not significant; therefore, resistance to change fully mediated the relationship between performance expectancy and performance expectancy. In addition, resistance to change partially mediated the relationship between social influence and behavioral intentions (variance accounted for [VAF]=43.3%), between work overload and behavioral intentions (VAF=37.2%), and between role ambiguity and behavioral intentions (VAF=12.2%).

**Table 7:** Mediation analysis (mediating variable=resistance to change).

Path	Indirect effect (95% CI)	Direct effect	*P* value	Variance accounted for (%)
Performance expectancy→behavioral intentions to use	0.18 (0.11 to 0.26)	0.10	.19	N/A^a^
Social influence→behavioral intentions to use	0.12 (0.07 to 0.16)	0.15	.002	43.3
Work overload→behavioral intentions to use	−0.05 (−0.10 to −0.02)	−0.09	.03	37.2
Role ambiguity→behavioral intentions to use	−0.05 (−0.11 to −0.01)	−0.34	<.001	12.2

^a^N/A: not applicable.

To compare the mediating effects within the resource gain approach and resource depletion approach and between the 2 approaches, we estimated the difference in indirect effects between the 2 mediating approaches. There was no significant difference in the mediating effects of resistance to change within the resource gain approach or resource depletion approach (Table 8). Notably, the mediating effects of the resource gain approach were significantly stronger than those of the resource depletion approach.

**Table 8:** Difference analysis of mediation.

Difference	Estimate (95% CI)	*P* value
Difference between performance expectancy and social influence	0.065 (−0.005 to 0.142)	.11
Difference between work overload and role ambiguity	−0.005 (−0.073 to 0.057)	.91
Difference between (performance expectancy + social influence) and (work overload + role ambiguity)	0.202 (0.101 to 0.286)	<.001

#### Moderated Mediation Model Analysis

In addition to the mediation model, we examined the moderating role of age, sex, professional title, and use experience in the processes of resource gain and resource depletion. Because sex is a 0 to 1 variable, a grouping mediation model was used to test the moderating effect of sex. Other moderating variables were tested by constructing interactive items. We first showed that the moderated mediation model fits (Multimedia Appendices 4 and 5) follow the corresponding guidelines by Maslowsky et al [49]. On the one hand, the model fit of the grouping mediation model was appropriate. On the other hand, the Akaike information criterion value of the mediation model with interaction terms was greater than that of the model without interaction terms, and the chi-square values were less than 0.05, indicating that the moderating mediation model fit was appropriate.

Table 9 shows the moderating roles of age, sex, professional title, and use experience on the impact of the mediation effects. The results showed that medical practitioners with different characteristics were sensitive to different resource approaches. Participants of different ages showed different effects of performance expectancy (β=−.03; *P*=.001) and role ambiguity (β=−.01; *P*=.03) on resistance to change. Participants of different sexes showed different effects of work overload and role ambiguity on resistance to change but showed no significant differences in the effects of performance expectancy and social influence on resistance to change. In terms of professional title, in addition to the path of work overload on resistance to change and behavioral intentions, professional titles played a substantial moderating role in other resource approaches. Finally, participants with different use experiences were more sensitive to the impact of role ambiguity on their resistance to change (β=−.03; *P*=.04).

**Table 9:** Moderated mediation analysis.

Paths and moderating variables			Β		*P* value
**Path 1: performance expectancy→resistance to change→behavioral intentions to use**					
	Age	−0.027		.001	
	Sex	0.168		.14	
	Professional title	0.355		<.001	
	Use experience	−0.027		.18	
**Path 2: social influence→resistance to change→behavioral intentions to use**					
	Age	0.004		.43	
	Sex	0.054		.39	
	Professional title	−0.110		.02	
	Use experience	0.010		.43	
**Path 3: work overload→resistance to change→behavioral intentions to use**					
	Age	−0.001		.89	
	Sex	0.085		.03	
	Professional title	0.080		.09	
	Use experience	−0.023		.09	
**Path 4: role ambiguity→resistance to change→behavioral intentions to use**					
	Age	−0.014		.03	
	Sex	0.096		.02	
	Professional title	0.142		.003	
	Use experience	−0.027		.04	

## Discussion

### Principal Findings

In this study, a qualitative interview of 16 experts and a cross-sectional study of 593 medical practitioners in China were conducted to explore the 4 major predictors of medical practitioners’ resistance and adoption to use internet hospitals, namely performance expectancy, social influence, work overload, and role ambiguity, and to examine how different characteristics of medical practitioners influenced these mechanisms. The small amount of existing internet hospital–related research focuses on patient perceptions [9,19,20], positive practice effects [7,21,50], or observations of the current state of development [3-5,18,51]. To the best of our knowledge, this is the first in-depth study to explore both the resistance to and adoption of internet hospitals from the perspective of medical practitioners.

Most importantly, this study advocates alleviating resistance to change as an important prerequisite for the transition to internet hospitals, which is in line with the consensus in the field of information management [52-54]. In previous studies, resistance to change has been a central variable in predicting the intention to use artificial intelligence–based clinical diagnostic decision support systems, web-based assistants, wearable medical technologies, and mobile medical apps [10,55-57]. Although existing research has amply identified various types of users' resistance to change in the face of mobile health services that affect their intention to use them [22,56,58], there is still a lack of focus on medical practitioners. From the perspective of medical practitioners, this study found that resistance to change, as one of the ways in which resources are conserved, mediates the effects of performance expectancy, social influence, work overload, and role ambiguity on behavioral intentions to use internet hospitals.

Among the resource gain approaches, performance expectancy and social influence helped release negative resistance and ultimately increased behavioral intentions to use internet hospitals. Recent research has also shown that performance expectancy and social influence are key variables in predicting behavioral intentions to use web-based assistants [55], wearable health technologies [56], and other health information systems and technologies [59]. At the same time, performance expectancy and social influence are direct predictors of clinician resistance to electronic drug management systems [60]. Consistent with the findings of this study, medical practitioners who believe that internet hospitals could help them diagnose more accurately and increase productivity are less likely to resist and more likely to accept, especially younger and less-senior practitioners. In addition, the results of social influence reflect that the support of colleagues, supervisors, and the public around them would encourage medical practitioners to eliminate resistance and adopt internet hospitals positively. This social influence is particularly valued by more senior medical practitioners.

The impact of performance expectancy was relatively greater than that of social influence, reflecting the cost compensation and incentive evaluation mechanisms for internet hospitals. Currently, the fees of follow-up consultations in most internet hospitals are uniform, which cannot reflect the difference in value between different levels of medical practitioners [3]. Consequently, medical practitioners are highly sensitive to performance incentives; therefore, increasing performance expectancy is an important intervention to break their resistance to change.

Among the resource depletion approaches, medical practitioners’ resistance to change is mainly driven by work overload and role ambiguity, which ultimately reduces their behavioral intentions to engage in the internet hospital. Previous work has shown that work overload has had a substantial negative impact on work concentration and work-family relationships of doctors and nurses [61-64] and has led to burnout and intention to leave [65,66]. In a recent study, work overload was identified as one of the key factors preventing health professionals from engaging in health care coordination [67]. According to the COR theory, the dual tasks from web-based and offline consultations may squeeze the remaining time and energy of medical practitioners, inducing work overload that may hinder the adoption of internet hospitals. However, we found that medical practitioners with more experience using internet hospitals were able to avoid this problem to some extent.

In addition, the results of role ambiguity in this study reflect that the unclear pricing and reimbursement of web-based services make it more confusing for medical practitioners to perform their roles in different environments. Previous research has shown that role ambiguity substantially predicts employees' readiness for organizational change and that clarifying employees’ roles is an important step in facilitating successful change [68]. According to the findings of this study, in the internet hospital scenario, medical practitioners facing role ambiguity are more likely to resist the internet hospital and their behavioral intentions to use it are reduced. These negative effects mainly affect female medical practitioners, which may be related to their choice of patient-centered communication, therefore spending more time with their patients [69].

The impact of work overload was relatively greater than that of role ambiguity, suggesting that developers and managers of internet hospitals should focus on avoiding the excessive workload growth of medical practitioners, which is also the main source of technostress for other health information applications [70].

Overall, positive feedback from medical practitioners on resource gain was stronger than negative feedback caused by resource depletion. Therefore, to motivate medical practitioners to transit and sustain health care services in internet hospitals, the internet hospitals’ implementers, managers, developers, and policy makers are suggested to establish a benefit distribution mechanism that matches the business model, especially in the preimplementation phase.

### Practical Implications

Exploitation of the findings in this study can provide practical implications for the future development of internet hospitals in terms of top-level design and operational models in China.

Comparing all the predictors that influenced resistance to or adoption of the internet hospital explored in this study shows that the overall impact of performance expectancy was greatest for medical practitioners. Given that the current price charges of most internet hospitals continue to be based on the price of general outpatient physician services, they do not reflect the difference in the value of the physician’s labor. Therefore, converting and reflecting the value of performance of the web-based health care delivery system of the internet hospital for offline benefits are the most effective ways to motivate medical practitioners to use the internet hospital. In addition, social influence also plays a key role in the resistance to and adoption of internet hospitals by medical practitioners. As a result, medical practitioners expect internet hospitals to provide adequate social support and build a good social reputation. We believe that the creation of an internet hospital treatment model based on a team of experts will be a good opportunity to help more medical practitioners expand their social influence.

The frustration because of work overload and role ambiguity caused by internet hospitals reflects the rapidly expanding demand for web access to health care and the bottleneck of inefficient web-based doctor-patient communication. It has been shown that patients’ unreasonable expectations of web-based services can substantially increase the workload of medical practitioners [9]. Therefore, for countries or regions where human resources for health are scarce, an independent operations management team should be established as soon as possible to minimize the workload of medical practitioners outside of medical services. In the overall service provided by internet hospitals, the operations management team can identify and analyze the needs of the patient before the consultation, release and monitor the efficiency of the doctor-patient communication during the consultation, and handle and track the feedback and evaluation of the patient after the consultation.

Finally, this study explored the resource conservation mechanisms behind the resistance to and adoption of internet hospitals by medical practitioners of different sexes, ages, titles, and experience. This helps hospital managers to rationalize a mix of human resources for health in internet hospitals. According to the results of this study, medical practitioners with lower and higher titles were more concerned about performance incentives and social encouragement, respectively. According to the results of this study, medical practitioners with lower titles and higher titles were more concerned about performance incentives and social encouragement, respectively. In addition, in the face of the clear division of labor expected of younger, female medical practitioners, prior experience of using internet hospitals will effectively mitigate the negative effects of role ambiguity. Therefore, understanding the structure and needs of different medical practitioners can help with prioritizing hospital incentives appropriately and maximizing their economic benefits. In countries and regions where there is a significant gap between general practitioners and family physicians, placing the right medical practitioners in internet hospitals can not only meet the increasing demand for internet treatment from patients but also help to ensure and improve the quality of health services provided by medical practitioners.

### Limitations and Future Research

This study has several limitations. First, the role of cross-cultural factors in resistance to change and behavioral intention to use internet hospitals has not been considered, which restricts the generalizability of the findings, particularly in high-income countries. Although the concept of internet hospitals may not be explicitly defined in other countries, various forms of internet hospitals still exist, such as Amwell in the United States. Future research is necessary to investigate and explore the operational models of various types of internet hospitals that may emerge in each country, from the multisubject perspective of patients, medical practitioners, and hospital administrators.

Second, this study does not extensively assess the actual resistance to using internet hospitals versus the actual use of internet hospitals because of the relatively short time after the recognition of internet hospitals and the ongoing preimplementation stage in many regions. As the scale of internet hospitals grows, future studies will be able to more conveniently collect data on the adoption of internet hospitals by patients as well as medical practitioners, which will help in the exploitation of research findings.

Third, the investigated variables were inherently correlated and based on a single source, thereby precluding any conclusions regarding causality. In particular, the snowball sampling method in the qualitative phase of this study is prone to sampling bias or self-selection bias, which undermines the credibility and comparability of the findings. Although we attempted to improve the representativeness and reliability of the sample by covering a broader group, including doctors, hospital administrators, government managers, and academicians, given the rapid pace of development of internet hospitals, we recommend that future studies uncover the dynamic changes in factors influencing resistance to or adoption of internet hospitals through a more targeted and multiple wave-based sampling method.

### Conclusions

This study provides an understanding of medical practitioners’ resistance to and adoption of the transition to the internet hospitals using a mixed methods research approach. On the basis of the combined results of qualitative interviews and empirical estimates, medical practitioners’ performance expectancy and social influence are the main sources of resource gains, whereas work overload and role ambiguity are the main sources of resource depletion. At the same time, medical practitioners of different ages, sexes, professional titles, and experience of use show different levels of sensitivity to different predictors. These insights will aid internet hospitals’ developers, implementers, and managers to better understand why medical practitioners resist using internet hospitals and how to facilitate the design and deployment of future practice strategies for internet hospitals.

## Appendix

Multimedia Appendix 1 Definition of constructs in the Unified Theory of Acceptance and Use of Technology and technostress framework.

Multimedia Appendix 2 Measurement items and loadings for quantitative study.

Multimedia Appendix 3 Mediation model fit.

Multimedia Appendix 4 Grouping mediation model fit for sex.

Multimedia Appendix 5 Moderated mediation model fit.

## Abbreviations

COR: conservation of resources
UTAUT: Unified Theory of Acceptance and Use of Technology
VAF: variance accounted for

## Data Availability

The data sets generated and analyzed in this study are available from the corresponding author upon reasonable request.
