# Supplementary material for: Investigating Factors Influencing Medical Practitioners’ Resistance to and Adoption of Internet Hospitals in China: Mixed Methods Study
Source: J Med Internet Res. 2023 Jul 31;25:e46621. doi: 10.2196/46621 (PMC10425818; doi:10.2196/46621)
Supplement: Multimedia Appendix 1 [file jmir_v25i1e46621_app1.docx]

**Multimedia Appendix 1.** Definition of constructs in the Unified Theory of Acceptance and Use of Technology Model and technostress framework.

| Constructs | | Definition |
| --- | --- | --- |
| **Unified Theory of Acceptance and Use of Technology Model (Venkatesh, Morris, Davis, & Davis)[29]** | | |
|  | Performance expectancy | The extent to which individuals perceive that the use of internet hospital helps to enhance the effectiveness of their work. |
|  | Effort expectancy | The extent to which individuals perceive ease of using internet hospital. |
|  | Social influence | The extent to which individuals perceive that their use of internet hospital is influenced by the surrounding environment. |
|  | Facilitating conditions | The extent to which individuals perceive the facilities needed to support the use of internet hospital. |
| **Technostress Framework (Ayyagari, Grover, & Purvis)[34]** | | |
|  | Work overload | Perception that using internet hospital exceeds an individual’s capability or skill level. |
|  | Invasion of privacy | Perception that individual’s privacy has been compromised. |
|  | Role ambiguity | Unpredictability of the consequences of one’s role performance and lack of information needed to perform the role. |
|  | Work-family conflict | An individual’s perceived conflict between the demands of work and family. |
