# Supplementary material for: Investigating Factors Influencing Medical Practitioners’ Resistance to and Adoption of Internet Hospitals in China: Mixed Methods Study
Source: J Med Internet Res. 2023 Jul 31;25:e46621. doi: 10.2196/46621 (PMC10425818; doi:10.2196/46621)
Supplement: Multimedia Appendix 2 [file jmir_v25i1e46621_app2.docx]

**Multimedia Appendix 2.** Measurement items and loadings for quantitative study.

| Constructs and related indicators | | Loading |
| --- | --- | --- |
| **Behavioral intention to use** | |  |
|  | 1. I intend to invest my time and effort in the internet hospitals. | 0.90 |
|  | 2. I intend to increase my use of the internet hospitals in the foreseeable future. | 0.90 |
|  | 3. I intend to switch from the current way of working to the internet hospitals. | 0.91 |
| Resistance to change | |  |
|  | 1. I don’t want the internet hospitals to change the way I care for patient processes. | 0.89 |
|  | 2. I don’t want the internet hospitals to change the way I make clinical decisions. | 0.89 |
|  | 3. I don’t want the internet hospitals to change the way I interact with other people on my job. | 0.86 |
|  | 4. Overall, I don’t want the internet hospitals to change the way I currently work. | 0.76 |
| Performance expectancy | |  |
|  | 1. I would find the internet hospitals useful in my job. | 0.89 |
|  | 2. If I use the internet hospitals, I will increase my chances of getting a salary increase or promotion. | 0.93 |
|  | 3. Using the internet hospitals enables me to accomplish tasks more quickly. | 0.88 |
|  | 4. Using the internet hospitals increases my productivity. | 0.76 |
| Social influence | |  |
|  | 1. People who are important to me think that I should use the internet hospitals. | 0.90 |
|  | 2. People who influence my behavior think that I should use the internet hospitals. | 0.91 |
|  | 3. The senior management of this hospital has motivated me in the use of the internet hospitals. | 0.88 |
| Work overload | |  |
|  | 1. Internet hospitals create many more requests, problems, or complaints in my job than I would otherwise experience. | 0.90 |
|  | 2. I feel busy or rushed due to internet hospitals. | 0.94 |
|  | 3. I feel pressured due to internet hospitals. | 0.92 |
| Role ambiguity | |  |
|  | 1. I am unsure whether I have to deal with internet hospitals problems or with my other work activities. | 0.86 |
|  | 2. I am unsure what to prioritize: dealing with internet hospitals problems or my other work activities. | 0.92 |
|  | 3. I cannot allocate time properly for my work activities because my time spent on internet hospitals -activities varies. | 0.92 |
|  | 4. Time spent resolving internet hospitals problems takes time away from fulfilling my other work responsibilities. | 0.89 |
