# Supplementary material for: Investigating Factors Influencing Medical Practitioners’ Resistance to and Adoption of Internet Hospitals in China: Mixed Methods Study
Source: J Med Internet Res. 2023 Jul 31;25:e46621. doi: 10.2196/46621 (PMC10425818; doi:10.2196/46621)
Supplement: Multimedia Appendix 4 [file jmir_v25i1e46621_app4.docx]

**Multimedia Appendix 4.** Grouping mediation model fit for sex.

|  | χ^2a^ | *df^b^* | χ^2^ */df* | CFI^c^ | TLI^d^ | RMSEA^e^ |
| --- | --- | --- | --- | --- | --- | --- |
| Threshold for acceptance fit | N/A^f^ | N/A^f^ | ＜5 | ＞0.900 | ＞0.900 | ＜0.080 |
| Model fit | 753.7 | 378 | 1.9 | 0.963 | 0.959 | 0.058 |
| ^a^ χ^2^ =chi-square  ^b^ *D.F.=*degrees of freedom  ^c^ CFI: Comparative Fit Index  ^d^ TLI: Tucker-Lewis Index  ^e^ RMSEA: Root Mean Square Error of Approximation  ^f^ N/A: Not applicable | | | | | | |
