# Supplementary material for: Investigating Factors Influencing Medical Practitioners’ Resistance to and Adoption of Internet Hospitals in China: Mixed Methods Study
Source: J Med Internet Res. 2023 Jul 31;25:e46621. doi: 10.2196/46621 (PMC10425818; doi:10.2196/46621)
Supplement: Multimedia Appendix 5 [file jmir_v25i1e46621_app5.docx]

**Multimedia Appendix 5.** Moderated mediation model fit.

| Moderating variable and index | | With interaction terms | Without interaction terms | Test value | Analysis |
| --- | --- | --- | --- | --- | --- |
| **Age** | |  |  |  |  |
|  | AIC^a^ | 23069.283 | 23064.706 | 4.577 | Pass |
|  | H0 Value | -11438.642 | -11432.353 | χ^2^=0.014 | Pass |
|  | Number of free parameters | 96 | 100 |  |  |
| **Professional title** | |  |  |  |  |
|  | AIC^a^ | 23060.302 | 23021.169 | 39.133 | Pass |
|  | H0 Value | -11434.15 | -11410.59 | χ^2^=0.000 | Pass |
|  | Number of free parameters | 96 | 100 |  |  |
| **Use experience** | |  |  |  |  |
|  | AIC^a^ | 23069.007 | 23043.014 | 25.993 | Pass |
|  | H0 Value | -11438.503 | -11421.507 | χ^2^=0.000 | Pass |
|  | Number of free parameters | 96 | 100 |  |  |
| ^a^ AIC: Akaike information criterion | | | | | |
